# The diagnostic utility of retroperitoneoscopic tissue biopsy for unresectable retroperitoneal lesions excluding urogenital cancers

**DOI:** 10.1186/s12957-019-1581-0

**Published:** 2019-02-18

**Authors:** Makito Miyake, Shinji Fukui, Daisuke Gotoh, Yoshiaki Matsumura, Shoji Samma, Yoshihiro Matsumoto, Hitoshi Momose, Shunta Hori, Shuji Watanabe, Takuya Owari, Yosuke Morizawa, Yoshitaka Itami, Yasushi Nakai, Takeshi Inoue, Satoshi Anai, Kazumasa Torimoto, Katsuya Aoki, Nobumichi Tanaka, Kiyohide Fujimoto

**Affiliations:** 1 Department of Urology, Nara Medical University, 840 Shijo-cho, Kashihara, Nara, 634-8522 Japan; 2 Department of Urology, Yamato Koriyama Hospital, 1-62 Asahii-cho, Yamato Koriyama, Nara, 639-1013 Japan; 3 Department of Urology, Nara Prefecture General Medical Center, 2-897-5 Shichijo-nishi-cho, Nara, Nara, 630-8581 Japan; 4 Department of Urology, Hoshigaoka Medical Center, 4-8-1 Hoshigaoka, Hirakata, Osaka, 573-8511 Japan; 5 Department of Urology, Saiseikai Chuwa Hospital, 323 Abe, Sakurai, Nara, 633-0054 Japan

**Keywords:** Retroperitoneal tumor, Retroperitoneoscopy, Biopsy, Complication, Urinoma

## Abstract

**Background:**

Retroperitoneal tumors are an uncommon disease known to consist of a diverse group of benign and malignant neoplasms. Treatment of unresectable retroperitoneal lesions requires pathological diagnosis. Here, we report the utility and safety of retroperitoneoscopic biopsy for unresectable retroperitoneal lesions excluding urogenital cancers.

**Methods:**

We analyzed 47 patients consisting of 23 (49%) and 24 (51%) cases that underwent retroperitoneoscopic tissue biopsy and open biopsy, respectively. The clinicopathological features, including postoperative complications, were compared between the two groups.

**Results:**

Tumor pathology was diagnosed successfully with a single operation in all patients. Malignant pathology (68%) was more common than benign pathology (32%). The most common pathology was malignant lymphoma, which accounted for about 50% of all cases. There was no significant difference with respect to the age, sex, tumor size, presence of tumor-related symptom, histopathology, operative time, and complications. Three (13%) of 23 patients in the retroperitoneoscopic biopsy group received percutaneous needle biopsy before laparoscopic excisional biopsy because the evaluation of needle cores failed to confirm subclasses of diagnosed pathologies. One patient was converted to open surgery just after the initiation of operation due to severe adhesion of adjacent structures. We had two cases with iatrogenic urinoma due to ureteral injury after retroperitoneoscopic biopsy.

**Conclusions:**

We conclude that retroperitoneoscopic biopsy is a safe and useful tool for benign and malignant retroperitoneal lesions, in comparison to open biopsy. It is critical to carefully examine the preoperative imaging for the location of tumors, especially those close to the renal pelvis and ureter.

## Introduction

Retroperitoneal tumors are an uncommon disease known to consist of a diverse group of benign and malignant tumors. According to a comprehensive review, primary retroperitoneal tumors account for 0.1–0.2% of all malignancies in the body, and 80–90% of all primary retroperitoneal tumors are pathologically malignant [1]. Retroperitoneal lesions represent expansive growth or infiltrative growth to the surrounding vital organs, muscles, and vessels, such as the kidney, ureter, psoas major muscle, vertebral, abdominal aorta, and inferior vena cava. The best therapeutic option is a surgical removal accompanied with extended resection of margins and adjacent organs, especially for retroperitoneal soft tissue sarcomas [2–5]. Several surgical techniques and advancements in open, transperitoneal laparoscopic, retroperitoneoscopic, and robot-assisted laparoscopic surgery have been introduced and reported for the management of retroperitoneal tumors [2, 6–9].

Due to the unique anatomy of the retroperitoneum, retroperitoneal tumors tend to be substantially enlarged, widely spread, and multiply with no or minimal symptoms. According to the NCI Dictionary of Cancer Terms, “unresectable” is defined as being unable to be removed with surgery due to spreading to the tissues around the primary lesion. A subset of patients has surgically unresectable retroperitoneal lesions, which are frequently indicated for tissue biopsy by means of computed tomography (CT)-guided needle biopsy, ultrasound-guided needle biopsy, or laparoscopic biopsy [10–12]. “Undetectable lesion” is defined as tumors which are unable to be removed with surgery due to its size, location, and/or expansion to surrounding organs. Although image-guided needle core biopsy or fine-needle aspiration (FNA) is a noninvasive and inexpensive procedure, one of the limitations is that a pathological evaluation of needle biopsy sometimes fails to confirm subclasses of lymphoma, sarcoma, and other neoplastic diseases due to insufficient amount of tissue specimens [13, 14], which could cause inappropriate treatment and delayed interventions.

Given these, we have extensively performed retroperitoneoscopic tissue biopsy aimed at accurate pathological diagnosis of surgically unresectable retroperitoneal lesions. In contrast to surgical approaches, reports on retroperitoneoscopic tissue biopsy have been very limited. Here, we address the feasibility and safety of this procedure for unresectable retroperitoneal lesions excluding urogenital cancer through a multicenter collaborative retrospective study.

## Methods

### Data collection

This study was approved by the ethics committee of the Nara Medical University, and all participants provided informed consent (reference ID: 1256 and 1594). The analyses and reporting of this study were conducted according to the Strengthening the Reporting of Observational Studies in Epidemiology (STROBE) guidelines, by using the checklist for observational studies [15].

Among patients diagnosed with retroperitoneal lesions excluding urogenital cancer (kidney cancer, urothelial cancer, prostate cancer, and testicular cancer) between 2001 and 2017 in our collaborative group hospitals, those receiving needle core biopsy were excluded from this study. Out of 47 patients, 23 (49%) and 24 (51%) underwent retroperitoneoscopic tissue biopsy and open biopsy, respectively. The clinicopathological data included age, sex, estimated tumor size, tumor-related symptoms, histopathology, operative time, pneumoperitoneum time, and postoperative complications (Table 1). The patients were followed up by routine blood examination and imaging examination depending on the histopathology of the tumor. Complications associated with tissue biopsy were objectively evaluated using the Clavien-Dindo classification system [16]. This system consists of seven grades (I, II, IIIa, IIIb, IVa, IVb, and V).

**Table 1:** Characteristics of 47 patients undergoing retroperitoneoscopic tissue biopsy or open biopsy for unresectable retroperitoneal lesion

Variables		Total cases	Type of tissue biopsy		*P* value
			Retroperitoneoscopy	Open	
No. of cases		47 (100%)	23 (49%)	24 (51%)	–
Age (year old)	Mean ± SD	63 ± 10	64 ± 10	62 ± 11	0.49 ^†^
	Median (range)	65 (40–79)	66 (40–79)	64 (43–78)	
Sex	Male	39 (83%)	21 (91%)	18 (75%)	0.24 ^‡^
	Female	8 (17%)	2 (9%)	6 (25%)	
Estimated tumor size (cm)	Mean ± SD	6.8 ± 4.5	5.7 ± 4.2	7.9 ± 4.7	0.07 ^†^
	Median (range)	5.4 (1.3–17.8)	4.2 (1.5–17.3)	8.0 (1.3–17.8)	
Tumor-related symptom^#^	None	30 (64%)	13 (56%)	17 (71%)	0.30 ^‡^
	Back pain	7 (15%)	6 (26%)	1 (4%)	
	Abdominal pain	5 (11%)	2 (9%)	3 (13%)	
	Nausea	3 (6%)	2 (9%)	1 (4%)	
	Leg edema	2 (4%)	1 (4%)	1 (4%)	
	Neurological symptom	1 (2%)	1 (4%)	0 (0%)	
	Palpable mass	1 (2%)	0 (0%)	1 (4%)	
Histopathology	Malignant lymphoma	23 (49%)	13 (56%)	10 (42%)	0.53 ‡
	Lymphoproliferative disease	8 (17%)	5 (22%)	3 (13%)	
	Metastatic lymph node	6 (15%)	2 (9%)	4 (17%)	
	Sarcoma	3 (6%)	1 (4%)	2 (8%)	
	Other benign lesion	7 (15%)	2 (9%)	5 (21%)	
Operative time (min)	Mean ± SD	169 ± 101	169 ± 57	170 ± 137	0.31 ^†^
	Median (range)	147 (16–388)	150 (80–326)	126 (16–388)	
Pneumoperitoneum time (min)	Mean ± SD	–	131 ± 63	–	–
	Median (range)	–	111 (69–278)	–	
Postoperative complication^##^	No	42 (89%)	19 (83%)	23 (96%)	0.14 ^‡^
	Retroperitoneal urine leakage	2 (4%)	2 (9%, grade IIIa)	0 (0%)	
	Persistent lymphorrhea	2 (4%)	2 (9%, grade II)	0 (0%)	
	Ileus	1 (2%)	0 (0%)	1 (4%, grade II)	

*SD* standard deviation

^#^Some patients had multiple symptoms

^##^Grading according to the Clavien-Dindo classification system [16]

^†^Mann–Whitney *U* test

^‡^Chi-square test or Fisher’s exact test

### Surgical procedure for retroperitoneoscopic tissue biopsy

**Fig. 1:**
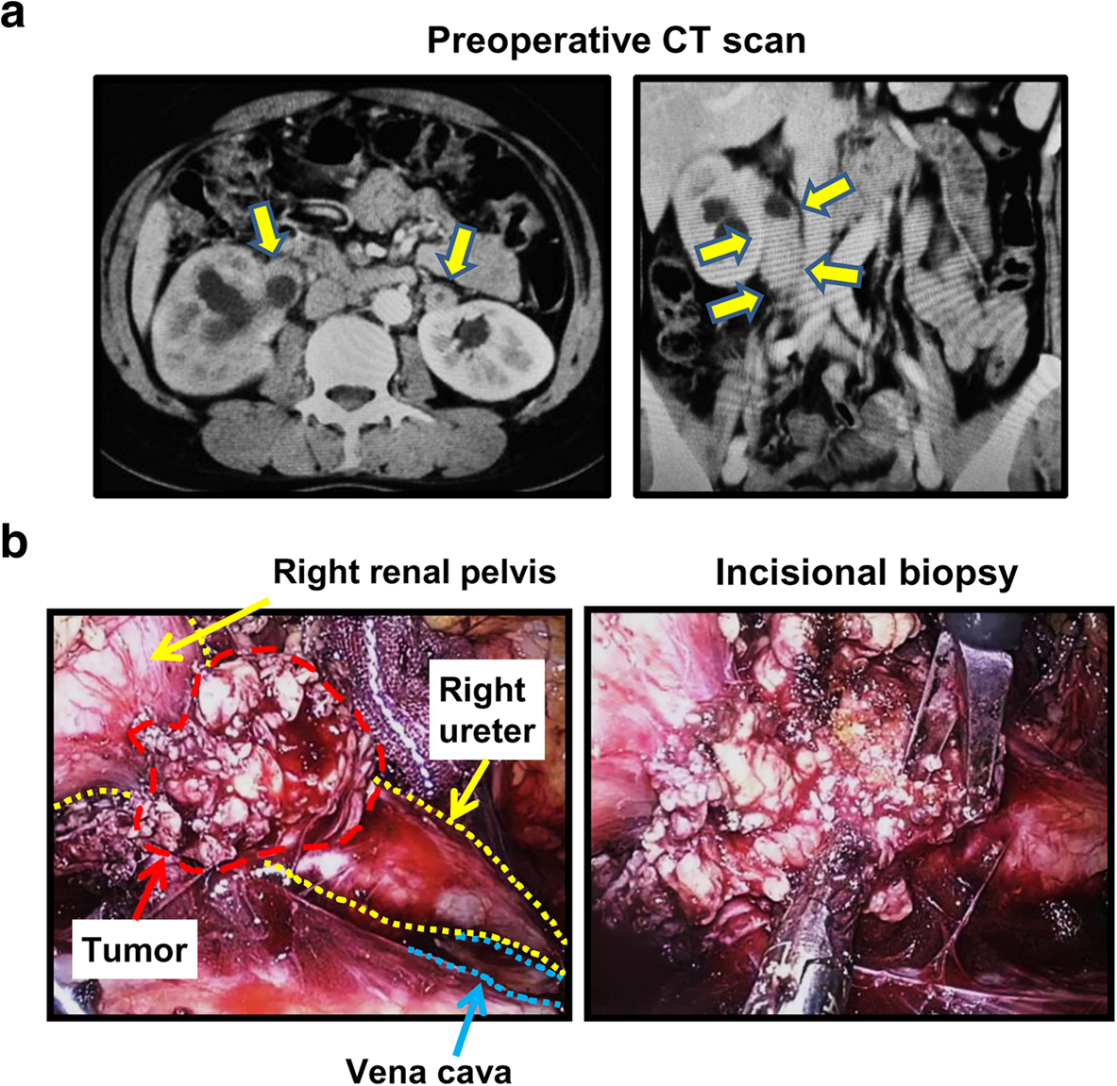
A representative case undergoing retroperitoneoscopic tissue biopsy. **a** Contrast-enhanced CT scan of a 39-year-old woman demonstrates extra-urinary tract lesions of bilateral ureters, which results in hydronephrosis. There are other multiple space-occupying lesions around the abdominal aorta and the inferior vena cava. **b** Intraoperative finding of the retroperitoneoscopic tissue biopsy. Tumors and their surrounding organs and structures are identified in order to make tissue biopsy easy and safe. A couple of tumor blocks are resected using cold scissors and removed through a small retrieval pouch. This case was pathologically diagnosed IgG4-related fibrosis

After induction of general anesthesia, each patient was placed in the lateral decubitus position for both retroperitoneoscopic biopsy and open biopsy. For retroperitoneoscopic biopsy, we took a conventional retroperitoneal approach with three or four ports (flexible endoscope, left-hand, right-hand, and auxiliary port) as previously described [17]. The retroperitoneal cavity was dilated with a retroperitoneal balloon and maintained with a high pressure (8 mmHg) of CO_2_. A representative case with bilateral extra-ureteral lesions is shown in Fig. 1. After retroperitoneal lesions were identified based on preoperative images (Fig. 1a), tumors were mobilized from surrounding organs and structures as much as possible to facilitate safe tissue biopsy (Fig. 1b). A couple of tumor blocks were resected using laparoscopic monopolar scissors (e.g., AESCULAP® laparoscopic instruments) without coagulation and removed into a small retrieval pouch for formalin fixation and freezing of specimens. We did not use needle biopsy instruments for retroperitoneoscopic tissue biopsy. We carefully provided hemostasis to the resected surface using electrocoagulation and hemostatic agents such as TachoSil® and SURGICEL®. A drainage tube was placed in the resection field.

### Surgical procedure for open tissue biopsy

The surgical incision and procedure varied depending on the surgeons. The surgery was based on retroperitoneal approach to the tumor. A couple of tumor blocks were resected using cold scissors for formalin fixation and freezing of specimens.

### Statistical analyses

The clinicopathological characteristics of the patients in this study were compared using a Mann–Whitney *U*, Chi-square, and Fisher’s exact test, whichever appropriate. PRISM software version 7.00 (GraphPad Software, Inc., San Diego, CA, USA) was used for statistical analyses. Statistical significance was set at *P* < 0.05, and all reported *P* values were two-sided.

## Results

We examined the difference in the clinicopathological characteristics between retroperitoneoscopic biopsy (*n* = 23) and open biopsy (*n* = 24) (Table 1). There was no significant difference with respect to the age, sex, estimated tumor size, presence of tumor-related symptom, histopathology, and operative time. Among the patients undergoing retroperitoneoscopic biopsy, one patient was converted to open surgery just after the initiation of operation due to severe adhesion to the adjacent structures. The estimated blood loss was less than 50 mL in most cases. One patient in open surgery received an autologous blood transfusion due to more than 500 mL blood loss.

The mean follow-up period was 28 months. Tumor pathology was diagnosed successfully with a single operation in all patients. In our cohort, malignant pathology (68%) was more common than benign pathology (32%). The most common pathology was malignant lymphoma, which accounted for about 50% of all cases. Out of 23 patients with retroperitoneal malignant lymphoma, 4 (17%) died of this disease. Of 15 patients (32%) diagnosed with benign tumors, 8 were diagnosed with pathologically lymphoproliferative disease with unknown cause and treated with steroid therapy after the biopsy. The remaining 7 patients consisted of 2 fibroadipose tissue, 1 chronic inflammation, 1 neurofibroma, 1 fibrous cyst, 1 cystic lymphangioma, and 1 schwannoma. Those 7 patients were followed up without any drug treatment and any second surgical treatment because tumors were benign. However, no clinical progression was observed during the postoperative follow-up.

Out of 23 patients in the retroperitoneoscopic biopsy group, 3 (13%) received CT-guided needle biopsy before laparoscopic excisional biopsy. The evaluation of needle cores failed to confirm subclasses of diagnosed pathologies due to insufficient amount or low quality of tissue specimens, which eventually caused delayed treatment interventions. Two of the 3 patients had follicular lymphomas and one had IgG4-related retroperitoneal fibrosis.

No perioperative mortality was observed. Although no statistical significance was found, 4 (18%) out of 23 retroperitoneoscopic biopsy group and 1 (4%) out of 24 open biopsy group experienced severe postoperative complications. We focused on 2 patients who had postoperative urine leakage due to urinary tract injury after retroperitoneoscopic biopsy.

**Fig. 2:**
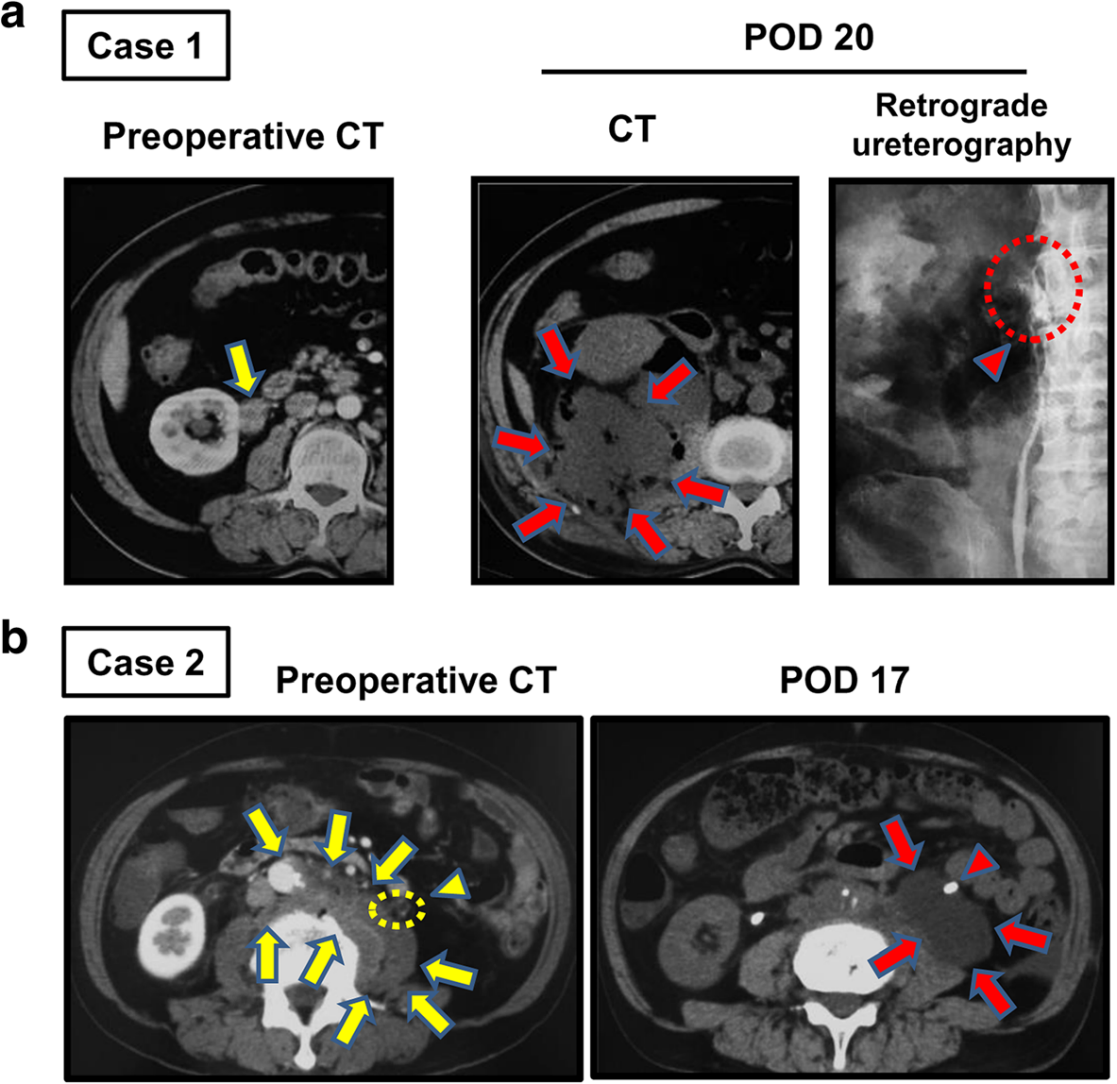
Two cases experiencing retroperitoneal urine leakage after retroperitoneoscopic tissue biopsy. **a** Case 1. Contrast enhanced CT scan of a 67-year-old man demonstrates extra-urinary lesions of the right ureter (yellow arrow), which results in ureteral obstruction and hydronephrosis. The CT scan on postoperative day (POD) 20 shows an infectious fluid storage in the retroperitoneal cavity (red arrows). Ureteral injury at the biopsy site is detected by the transurethral retrograde ureterography (red arrowhead). **b** Case 2. Contrast-enhanced CT scan of a 64-year-old man demonstrates retroperitoneal lesions involving aorta, inferior vena cava, common ileac arteries, and left psoas (yellow arrows). The left ureter is located close to the lesion (yellow dashed line and arrowhead). The CT scan on POD 17 shows a cystic urinoma in the retroperitoneal cavity (red arrows). The red arrowhead indicates the ureter with a ureteral stent

The first case was a 67-year-old man with asymptomatic retroperitoneal lesions involving the right renal pelvis and ureter, which was later revealed as diffuse large B-cell malignant lymphoma (Fig. 2a). A ureteral stent was placed for safety before retroperitoneoscopic biopsy. The biopsy was successfully performed, and the ureteral stent was removed 7 days after the biopsy. At postoperative day (POD) 20, he visited the emergency room with complaints of high fever and back pain. The CT scan demonstrated infectious urine leakage in the right-side retroperitoneal cavity with suspicion of ureter injury at the biopsy site (Fig. 2a). He recovered after percutaneous nephrostomy (PNS) was placed to drain infectious fluid. About a year from the PNS placement, complete remission of retroperitoneal malignant lymphoma was achieved by intensified and long-term systemic chemotherapy. After the PNS was removed, he received an endoscopic surgery for ureteral stricture caused by the ureteral injury.

The second case was a 64-year-old man with asymptomatic retroperitoneal lesions involving the aorta, inferior vena cava, common ileac arteries, and left psoas, ranging from the level of the second lumbar vertebra to the sacrum (Fig. 2b). As the tumor was revealed to be IgG4-related retroperitoneal fibrosis, he started taking steroid tablets (40 mg/day). At POD 17, he visited the emergency room with complaints of high fever and left flank pain. The CT scan demonstrated an urinoma in the left-side retroperitoneal cavity with strong suspicion of ureteral injury (Fig. 2b). He received placement of a ureteral stent and antibiotics. In spite of the shrinkage of urinoma with time, a high fever continued. Therefore, PNS was placed in the left renal pelvis. Five months after the retroperitoneoscopic biopsy, the PNS was removed.

## Discussion

The present retrospective study examined the unresectable retroperitoneal lesions undergoing the retroperitoneoscopic tissue biopsy. Because primary retroperitoneal tumors and lesions are a rare disease, there was a need to conduct a multicenter-based collaborative study (among five institutes) to assess a sufficient number of cases. A total of 47 cases consisting of 23 undergoing retroperitoneoscopic tissue biopsy and 24 undergoing open biopsy were compared with respect to the selected clinicopathological features. There was a trend that patients undergoing open biopsy had larger tumors (*P* = 0.07, median 4.2 cm vs. 8.0 cm). Contrary to our expectations, patients undergoing open biopsy did not have tumor-related symptoms more frequently at the time of diagnosis (*P* = 0.30, 56% vs. 71%) and the open procedure did not require a longer operative time (*P* = 0.31, median 150 min vs. 126 min). Based on our findings, the retroperitoneoscopic tissue biopsy seems to be the same as the conventional open biopsy in terms of diagnostic utility and safety.

Open surgery is a conventional modality for resection and tissue biopsy; however, this approach may result in postoperative pain, a prolonged recovery time, and long-term hospitalization. Recent advances in laparoscopic techniques and devices have enabled a safe and minimally invasive procedure by visual magnification providing adequate biopsy specimens. Retroperitoneal approach enables direct access to lesions without manipulation of abdominal organs and decreases the risk of injury to neighboring organs. A previous report demonstrated the utility and safety of retroperitoneoscopic resection for paragangliomas, schwannomas, and adrenals with distinct advantages, including direct access to tumors, low intraperitoneal interference, precise dissection, and minimal invasiveness [18–20]. We should carefully consider the application of laparoscopic surgery in patients with malignant retroperitoneal tumor. A case report from Japan demonstrated a port-site recurrence after laparoscopic surgery for a liposarcoma [21]. The utility of the retroperitoneoscopic resection technique for sarcomas remains controversial. We removed the tumor specimens through a small retrieval pouch immediately after incision biopsy in order to prevent dissemination of tumor cells. No patients experienced a port-site recurrence in our cohort.

Back pain, abdominal pain, anorexia, nausea, and edema are frequently observed symptoms associated with this disease. Because of a recent prevalence of health check-ups and advancement in imaging technologies, the number of patients diagnosed with asymptomatic retroperitoneal tumors seems to be increasing. However, there are still many patients who had multiple and huge tumors in the retroperitoneal cavity at the first presentation. CT scan and magnetic resonance imaging (MRI) can inform the physicians of estimated tissue composition in benign and malignant lesions, tumor location and multiplicity, and invasion to neighboring structures. According to the radiographic information, physicians should decide the resectability and follow an optimal treatment strategy. Although the treatment modality of unresectable tumors is limited, the most essential information is pathological diagnosis including the subtypes of malignancy. All the 47 patients in our cohort were successfully diagnosed with a single operation (Table 1). Other noninvasive diagnostic methods, including needle core biopsy and FNA, sometimes fail to confirm accurate subclasses of malignancy due to insufficient amount of tissue specimens [22–24]. Some malignant neoplasms require an adequate/sufficient amount of tissue specimens for specialized studies such as immunohistochemical analysis, flow cytometry, fluorescence in situ hybridization, and genetic rearrangement testing. Moreover, percutaneous biopsy of retroperitoneal lesions can be difficult and unsafe owing to the tumor size and adjacent organs such as the intestines and major blood vessels [10]. Laparoscopic incisional biopsy is one of the most reliable and accurate diagnostic methods in certain patients.

Ultrasonography-guided FNA is a minimally invasive sampling technique. Many papers including case reports have shown its safety, clinical usefulness, and limitation for diagnosing retroperitoneal tumors such as schwannoma [25], lipoblastoma [26], histiocytic sarcoma [27], liposarcoma [28], and abscess. Most of them are not able to be diagnosed solely from image findings. Diagnostic accuracy of FNA varies among types of tumor. However, diagnostic accuracy of endoscopic ultrasound-guided FNA followed by cytomorphologic features and immunocytochemistry analysis is only 66.7% in schwannoma [25]. Out of nine patients with lipoblastoma, the FNA diagnosis was inconclusive due to hypocellularity in one patient, and a diagnosis of benign lipomatous tumor was made in another patient [26]. Regarding histiocytic sarcoma, the diagnosis with FNA alone is extremely challenging [27]. Overall, the FNA should be accompanied with molecular biology techniques detecting gene alteration or rearrangement for achieving a high diagnostic accuracy.

The maximal effort should be made to reduce the risk of injury of neighboring structures. Iatrogenic ureteral injuries are a relatively common complication in intrapelvic surgery and radiotherapy [29]. Although gynecologic surgery accounts for the majority (64–82%) of the approaches used for ureteral injuries, urologic intervention, including ureteroscopy, lymphadenectomy, and urinary diversion, accounts for 11–30% [30]. More than 65% of urinary injuries are detected postoperatively [29]. Early diagnosis and treatment are vital for satisfactory outcomes. A retrograde pyelography allows both the diagnosis and placement of an indwelling ureteral stent that recovers urinary drainage from the renal pelvis to the bladder. If a retrograde pyelography is impossible, a PNS placement is combined with an attempt to place an indwelling stent in an antegrade fashion. For short defects < 2.5 cm, the placement of an indwelling ureteral stent is an effective treatment, which can be removed after 2–6 weeks [29]. The placement of a ureteral stent can achieve a success rate of < 70% for ureteral realigning [31, 32]. We had two cases with iatrogenic urinoma caused by ureteral injury (Fig. 2). Urinary injury was detected postoperatively on PODs 17 and 20. For case 1, the ureteral stent was preoperatively placed for safety and removed at POD 7. Placement of a ureteral stent before tissue biopsy procedure and a longer placement of the ureteral stent after retroperitoneoscopic biopsy may be preferred for safety. In both cases, the biopsy site was closed to the ureter and we did not notice ureter injury. We should take a great caution for the biopsy of retroperitoneal lesions close to ureters.

The present study has several limitations. The first is its retrospective nature with a potential selection bias. For example, the decision between retroperitoneoscopic and open approaches was made by physicians rather than randomization. Second, the data were from multiple institutes; therefore, surgery was not performed by a single surgeon. Third, there were missing data regarding postoperative pain such as visual analogue scale scores and patient-reported outcome data such as health-related quality of life, which could help confirm the true clinical value of the retroperitoneoscopic approach. Finally, the sample size is relatively small.

In conclusion, we reviewed our experience of diagnostic retroperitoneoscopic biopsy for unresectable retroperitoneal lesions excluding urogenital cancers. The comparison with the cohort of open biopsy revealed that retroperitoneoscopic biopsy is a safe and useful tool for benign and malignant retroperitoneal lesions. It is crucial to pay attention in the preoperative imaging concerning the tumor location, especially for tumors close to renal pelvis and ureter.

## Abbreviations

CT: Computed tomography
FNA: Fine-needle aspiration
PNS: Percutaneous nephrostomy
POD: Postoperative day
